# Bioactive Olivacine Derivatives—Potential Application in Cancer Therapy

**DOI:** 10.3390/biology10060564

**Published:** 2021-06-21

**Authors:** Beata Tylińska, Benita Wiatrak

**Affiliations:** 1 Department of Organic Chemistry, Faculty of Pharmacy, Wroclaw Medical University, 50-556 Wroclaw, Poland; 2 Department of Pharmacology, Faculty of Medicine, Wroclaw Medical University, 50-345 Wroclaw, Poland; benita.wiatrak@umed.wroc.pl

**Keywords:** olivacine, pyridocarbazole, cytostatic, S16020, antitumor, in vitro, in vivo

## Abstract

**Simple Summary:**

Olivacine is a compound isolated from the bark of *Aspidosperma olivaceum* (a tree found mainly in Southeastern Brazil) that shows multidirectional biological activity. The most important of them is the antiproliferative effect, important in anticancer therapy. This article reviews the literature on the results of research on olivacine and its derivatives carried out in cell laboratories, in preclinical studies in animals and clinical trials in humans. The described activities of these compounds were discussed by comparing the differences in their structure. The most important finding of this review is that some olivacine derivatives exhibit greater anticancer activity than doxorubicin (a commonly used anticancer drug).

**Abstract:**

Olivacine and its derivatives are characterized by multidirectional biological activity. Noteworthy is their antiproliferative effect related to various mechanisms, such as inhibition of growth factors, enzymes, kinases and others. The activity of these compounds was tested on cell lines of various tumors. In most publications, the most active olivacine derivatives exceeded the effects of doxorubicin (a commonly used anticancer drug), so in the future, they may become the main new anticancer drugs. In this publication, we present the groups of the most active olivacine derivatives obtained. In this work, the in vitro and in vivo activity of olivacine and its most active derivatives are presented. We describe olivacine derivatives that have been in clinical trials. We conducted a structure–activity relationship (SAR) analysis that may be used to obtain new olivacine derivatives with better properties than the available anticancer drugs.

## 1. Introduction

Pyridocarbazole derivatives, which are alkaloids, have become of interest to many scientists due to their biological activity. The two alkaloids, olivacine and its ellipticine isomer [1,2], showed marked antitumor activity [3,4]. Many laboratories worldwide are trying to modify the structure of the heterocyclic system of pyridocarbazole to obtain analogs with a better therapeutic index. Numerous publications on the subject indicate that the introduction of substituents at the C-1, N-2, C-9, C-11 positions of the pyrido[4,3-*b*]carbazole system will play a significant role in the pharmacological activity of the derivatives of the alkaloids in question. In vitro studies have shown that hydroxylation of the C-9 position of various ellipticine and olivacine derivatives increases cytostatic activity over the 9-methoxy derivatives, caused by a greater affinity of the compound for DNA-increased stabilization of the DNA–topo II complex. The mechanism of action and antitumor properties of olivacine are similar to ellipticine and are considered DNA intercalation and topoisomerase II inhibition [5,6,7,8,9,10]. The cytotoxic effect of ellipticine is also related to the impact on the p53 protein [11,12]. Ellipticine is covalently inserted into the DNA on the metabolic side by cytochrome P450 or peroxidase isoforms [13,14,15,16,17,18,19,20,21]. It should be emphasized that some olivacine derivatives, such as the compound S16020, showed a broad spectrum of antitumor activity and greater activity compared to the ellipticine derivatives and doxorubicin [22,23,24,25]. The mechanism of olivacine and ellipticine action is similar, but more publications concern ellipticine, which exhibits greater cytotoxicity than olivacine. However, research shows that olivacine derivatives are less toxic to normal NHDF cells (normal human dermal fibroblasts) than doxorubicin and ellipticine [26,27]. At the same time, it was observed that olivacine derivatives had a stronger effect on the p53 protein (one of the most important suppressors of tumor transformation) level than ellipticine. This article presents reports on olivacine and the most active olivacine derivatives (tested in vitro and in vivo). Structure–activity relationship (SAR) analysis may be used to obtain new olivacine derivatives with better properties than the available anticancer drugs.

## 2. Olivacine

**Figure 1:**
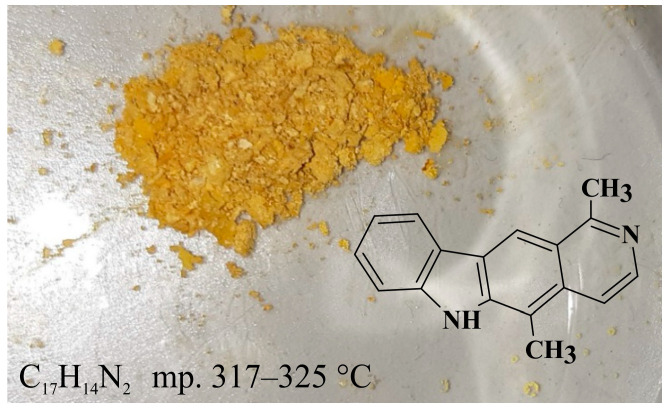
Olivacine (guatambuinine).

Olivacine **1** (1,5-dimethyl-6H-pyrido[4,3-*b*]carbazole) (guatambuinine) (Figure 1) is an alkaloid and was isolated for the first time from the bark of *Aspidosperma olivaceum* [2]—a tree found in Southeastern Brazil, Argentina, Paraguay and Bolivia [28]. *Aspidosperma olivaceum* is a photophilous deciduous tree, reaching a height of 25 m and a trunk diameter of 90 cm, with white flowers and fleshy fruit (drupe) [29]. Plants of the *Aspidosperma* order have traditionally been used in Brazilian diagnostics to combat fever and other ailments. The receptors behind these plants’ pharmacological action—anti-inflammatory, analgesic, antibacterial—are primarily opposed by monoterpene indole alkaloids. Anti-malarial extracts from these vegetation sources, traditionally obtained from bark decoctions, are used in folk medicine during malaria [30].

*A. olivaceum* exhibits the anti-malarial effect on quinine-sensitive and quinine-resistant clones of *P. falciparum,* reaching an IC_50_ value below 10 µg/mL [31]. Olivacine inhibits the growth of *P. falciparum* in vitro (IC_50_ = 1.2 µM). Noteworthy is the lack of toxic effects of olivacine even in administering high doses (up to 100 mg/kg/day) [32]. Research by Touaty and Simon shows that olivacine affects the growth of *E. coli*. In vivo tests indicate that low levels of olivacine have a much stronger effect on the inhibition of protein synthesis in *E. coli* bacteria than on DNA and RNA synthesis [33]. The interest in olivacine was due to the discovery in 1966 of its anticancer properties. The antitumor activity of olivacine prompted the development of new syntheses to prepare this alkaloid, as a large amount of the compound was needed for further biological tests [3]. To date, more than twenty methods of synthesizing olivacine have been described [34].

Olivacine derivatives that showed better anticancer activity have also begun to be synthesized. There are many possible ways to modify the olivacine structure by extending the side chain at positions C-1, N-5, and C-9 and changing the elements in the pyridocarbazole skeleton.

## 3. The Most Active Olivacine Derivatives

### 3.1. Structure–Activity Relationship (SAR) Analysis In Vitro

Structure–activity relationships are based on the discovery that the biological and physicochemical activity of chemical compounds depends on the structure of molecules. Due to this discovery, it can be expected that modification of molecules (replacement of one substituent with another, reduction or enlargement of the molecule) may result in compounds showing better activity or selectivity or will be characterized by better pharmacokinetics.

**Figure 2:**
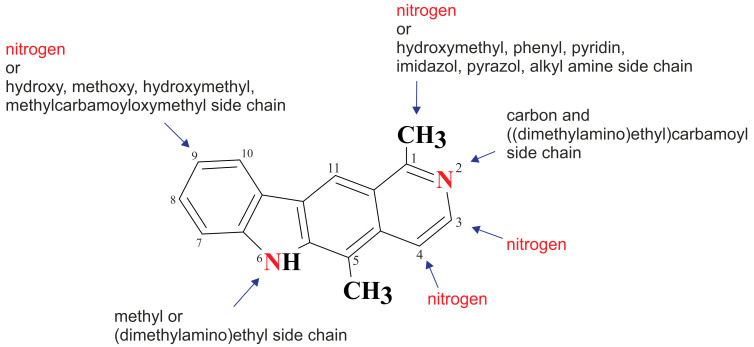
Modification of the structure of olivacine.

After reviewing the literature, we chose the most active olivacine derivatives described in Table 1. We compared compounds that were tested for in vitro cytotoxic activity (IC_50_ µM ± SD) on mouse leukemia (cell line L1210), non-small-cell lung cancer (cell line A549), and breast cancer (cell line MCF-7) cells. We can see how the anticancer activity changes, thereby analyzing olivacine derivative structures using the SAR method (Figure 2).

From the literature studies listed in Table 1, we can see that the cytotoxicity of olivacine **1** IC_50_ against L1210 tumor cells (murine leukemia) is 2.03 µM. At compound **3**, where the carbon at C-3 has been replaced with a nitrogen atom, the antitumor activity is comparable, IC_50_ = 1.79 µM (L1210), IC_50_ = 4.5 µM (MCF-7), to olivacine 1 activity. By introducing a hydroxyl moiety at the C-9 position of olivacine **1**, the activity increases to IC_50_ = 0.06 µM (L1210) for 9-hydroxyolivacine **2**. Based on Table 1, which describes the most active olivacine derivatives synthesized and described in the literature, it can be stated that the hydroxyl group significantly increases the antitumor activity because as many as **17** derivatives out of **29** described in Table 1 have this grouping. There is also a methoxy moiety (six compounds) at the C-9 position. However, it is this group that lowers the activity. Compound **4** (pazellipticine) has nitrogen deposited in place of carbon C-9; its activity is comparable to that of 9-hydroxyolivacine **2**, but here an increase in activity can be expected after introducing the ((diethylamino)propyl)amine moiety at the C-1 position. The importance of the hydroxyl group in the C-9 position can be seen in the activity of compounds **6** and **7**. Both compounds have a ((diethylamino)propyl)amino substituent in the C-1 position, differing only in structure with the substituent in C-9. Compound **6** has a hydroxyl group and IC50 = 0.02 µM (L1210) activity, and compound **7** has a hydroxyl group at the C-9 position and activity of IC_50_ = 1 µM (L1210). The best antitumor activity was shown by compound **9**, known in the literature as S16020, IC_50_ = 0.0041 µM (L1210), IC_50_ = 0.030 µM (A549), IC_50_ = 0.075 µM (MCF-7). This relationship has been demonstrated in clinical trials. Because its activity was exceptional, its derivatives were synthesized. Compound **8**, known in the literature as S30972-1, was obtained, with IC_50_ = 0.019 µM against L1210. Its structure differs from S16020 with a substituent at position C-9. Instead of a hydroxyl group at the C-9 position, it has pentanedioic acid; its IC_50_ = 0.019 µM against L1210 and is one order lower than S16020. Compound **11** was also obtained, a methylcarbamoyloxymethyl substituent in the C-9 position and an IC_50_ = 1.25 µM against A549. It can be seen that replacing the hydroxyl group at the C-9 position with another group reduces the antitumor activity. S16020 derivatives were also obtained, which had a phenyl introduced between the pyrido[4,3-*b*]calbazole moiety in the C-1 position and the ((dimethylamino)ethyl)carbamoyl moiety. Compound **19** has IC_50_ = 7.15 µM against L1210, IC_50_ = 8.19 µM against A549, and compound **20** has IC_50_ = 6.08 µM (L1210), IC_50_ = 8.25 µM (A549). It can be seen that the introduction of phenol caused a decrease in anticancer activity. Also introduced in this position was pyridine (compound No **23**) IC_50_ = 0.05 µM (L12010), IC_50_ = 0.095 µM (A549), IC_50_ = 0.23 µM (MCF-7), whose activity turned out to be very interesting. Compounds were also synthesized, which in the C-9 position of the pyrido[4,3-*b*]carbazole had a hydroxyl group, and in the C-1 position had pyridine, but no ((dimethylamino)ethyl)carbamoyl group; activity for compound **24**, IC_50_ = 0.9 µM (L1210), IC_50_ = 5.03 µM (A549), and for compound **26**, IC_50_ = 0.8 µM (L1210), shows a decrease in biological activity here. S16020 has also been modified to introduce nitrogen in place of carbon at the C-2 and C-4 positions. For compound **15**, IC_50_ = 0.010 µM (L1210). Nitrogen was introduced into compound **16** at the C-1 and C-4 positions, and the ((dimethylamino)ethyl)carbamoyl moiety was placed at the C-2 position. The activity for compound **16** was IC_50_ = 0.33 µM (L1210). Cytotoxicity turned out to be very interesting, especially for compound **15**. Among the most active olivacine derivatives described in Table 1, as many as 21 have a methyl group in the C-6 position, and one compound, No. **25**, has in the C-6 position dimethylaminoethyl, with IC_50_ = 1.5 µM (L1210), IC_50_ = 2.12 µM (A549). Compound **24**, comparable to compound **25**, having a methyl substituent in the C-6 position, has IC_50_ = 0.9 µM (L1210), IC_50_ = 5.03 µM, (A549).

Of interest are compounds **28** and **29**, which have 4-nitro-imidazole (compound **28**) and 4-nitro-pyrazole (compound **29**) at the C-1 position. The compounds were tested for antitumor activity under aerobic and anaerobic conditions. For compound **28** in normoxic conditions IC_50_ = 4.74 µM (A549), IC_50_ = 5.96 µM (MCF-7) and in hypoxic conditions IC_50_ = 0.57 µM (A549), IC_50_ = 0.69 µM (MCF-7). For derivative **29** in normoxic conditions IC_50_ = 30.5 µM, (A549), IC_50_ = 11.25 µM (MCF-7) and in hypoxic conditions IC_50_ = 0.65 µM (A549), IC_50_ = 0.81 µM (MCF-7). Compounds **28** and **29** seem to be very interesting because some cancers develop in anaerobic conditions, and there is a need for compounds that will selectively affect cancer tumors.

#### 3.1.1. 9-Hydroxyolivacine **2**

U. Schmidt et al. presented the synthesis and biological studies of olivacine derivatives [52]. They described 8-hydroxyolivacine, 9-hydroxyolivacine and 8-methoxyolivacine and 9-methoxyolivacine. The compounds were tested for growth inhibition of *Mycobacterium tuberculosis.* 9-methoxyolivacine showed significant inhibition, MIC90 = 1.5 µM and olivacine, MIC90 = 4.7 µM, which shows that olivacine and its derivatives have antitumor activity and show wider bioactivity. The authors rightly note that olivacine’s pharmacological potential and its derivatives are much less studied than that of ellipticine and its derivatives [52].

#### 3.1.2. Azo-Olivacine Derivatives **4**, **15**, **16**

Olivacine was modified by introducing nitrogen atoms into the pyrido[4,3-*b*]carbazole system instead of carbon atoms. They showed various anticancer effects.

Pazelliptine **4** (PZE) is a 9-aza derivative of olivacine [36], which exhibits greater antitumor activity and is less toxic than ellipticine [53]. Unlike C-9 ellipticine derivatives, which can be oxidized at the C-10 position and form harmful free radicals capable of alkylating proteins or nucleic acids, N-9 derivatives do not undergo such transformations. They are probably metabolized by oxidation of the nitrogen atom at the N-6 position [54]. Moreover, it seems that ESA is not active against DNA in isolated cell nuclei, probably due to the rapid metabolism of this compound [55].

3-Aza-olivia influenced cancer cells of the cervix (KB), lymphocytic leukemia (L-1210), ovary (SK-OV-3) and breast (MCF-7) only at a relatively high concentration of 3.16 µg/mL. In contrast, at lower concentrations, no dose-dependent effect was observed [56]. It seems that such a modification is not the best.

Studies on other S16020 **9** derivatives have shown that the cytotoxicity is influenced not only by the number of nitrogen atoms but also by the location. The pyrazinocarbazole derivative **16** has been found to exhibit weak cytotoxicity to tumor cells, while derivative **15** has similar cytotoxicity against L1210 murine leukemia cells and P388 leukemia to the parent compound S16020 9 [44].

### 3.2. SAR In Vivo

The in vivo evaluation of olivacine and its derivatives was performed on two experimental mouse models, L12010 leukemia and P388 leukemia. The therapeutic effect of drugs was measured as the percent increase in life span (% ILS) over controls, evaluated as follows: % ILS = (median survival time (MST) in treated) − (median survival time in controls)/(median survival time in controls) × 100 or % T/C = median survival time of treated/median survival time of control animals. From the data presented in Table 1, we can see that administering olivacine **1** to diseased mice at a dose of 250 mg/kg increased their survival by 35% (ILS), at a dose of 84 mg/kg 141% (T/C). Modifying the olivacine structure and replacing carbon at 9-C with nitrogen, and introducing a diethylaminopropylamine group at C-1 (compound **4**) resulted in an increase in survival to 85% (ILS) at a lower dose of 20 mg/kg. Compound **5** has such an alkylamino chain in the 1-position and the methoxy group in the 9-position at a dose of 15 mg/kg, 24% (ILS). Changing the moiety to hydroxyl at position 9 in compound **6** resulted in a dose reduction of 5 mg/kg and increased survival to 49.5%. Extending the alkyl chain and introducing a new CH_2_ group reduced the activity of compound **7** (20 mg/kg, 21% (ILS), 10 mg/kg, 16% (ILS)) compared to the very similar compound **5**. Compound **8** showed the best activity at doses of 160–320 mg/kg, 246->590% (T/C), 80 mg/kg, 427->582% (T/C). 1-((2-dimethylamino)ethyl)carbamoyl)-5,6-dimethyl-9-hydroxy-6*H*-pyrido[4,3-*b*]carbazole (S16020) **9** dose 90 mg/kg, 238% (T/C), 120 mg/kg 301% (T/C). After introducing the [(dimethylamino)ethyl]pyridine substituent in position 1 of the pyridocarbazole system, compound **23** was obtained, which at a dose of 40 mg/kg showed a therapeutic effect of 207% (T/C).

### 3.3. Clinical Trials

So far, the following ellipticine derivatives have been used in clinical trials: celiptium [57,58,59], datelliptium [60,61], retelliptine [61,62], elliprabin [61] and olivacine derivative: S16020 [25].

So far, the most active synthesized olivacine derivative is 9-hydroxy-5,6-dimethyl-1-(2-(dimethylamino)ethyl)carbamoyl)-6*H*-pyrido[4,3-*b*]carbazole **9**, known in the literature as S16020. It is an olivacine derivative containing a (2-(dimethylamino)ethyl)carbamoyl moiety in the C-1 position. In the N-6 position, it has a methyl group that blocks the pyrrole nitrogen atom and prevents oxidation of the 9-hydroxy group to the quinoid system responsible for the toxicity of the compound [63]. The derivative of pyrido[4,3-*b*]carbazole **9** showed significant activity in preclinical studies against the murine leukemia P388, melanoma B16, sarcoma M5076 and human colon C38 and breast cancer cells, ovary and lung [23,25,40,56,64,65,66,67,68]. Thanks to its activity comparable to cyclophosphamide [66] and doxorubicin [64,67] and relatively low toxicity, it was qualified for clinical trials [56,68]. S16020 was synthesized by R. Jasztold-Howorko, who published the synthesis and biological research of this compound in 1994 [40]. The compound has obtained patent protection in European countries, Japan, and the United States and has been subjected to clinical trials [56,69]. Unfortunately, the compound was not approved for further research. It turned out that, although the average survival time of patients treated with S16020 was slightly longer than that of patients treated with methotrexate, facial and tumor swelling as well as erythematous rash prevented the inclusion of the new olivacine derivative in the next phase of clinical trials [70]. However, only this derivative of olivacine has been so thoroughly researched, and it is the subject of the largest number of articles.

As a result of arylation of S16020 **9** with glutaric anhydride, the new compound S30972-1 **8** was obtained, which turned out to be more active in vivo and less toxic than doxorubicin and etoposide [71]. The study found that combination 8 is presumably a prodrug of S16020 **9** [72].

### 3.4. Influence on the Effectiveness of Radiotherapy

The derivative S16020 has also been studied in conjunction with radiation therapy. Combining ionizing radiation with intercalators [73] and inhibitors of topoisomerase II [22] often leads to an increase in its toxicity. This was demonstrated in studies using the HEP2 human tumor line and mice, comparing the effects of ionizing radiation in combination with S16020. Differences in the activity of topoisomerase II in tissues (a higher expression of topoisomerase II characterizes tumor cells compared to normal cells) cause the S16020 compound used alone to increase the cytotoxic effect of radiation during ionization, without increasing its toxicity to healthy tissues and susceptibility to fungal infections. The study also showed that the order of receiving the dose of radiation and S16020 (20 days apart or simultaneously) did not affect their action [74,75].

## 4. Mechanisms of Antitumor Activity of Olivacine and Olivacine Derivative

**Figure 3:**
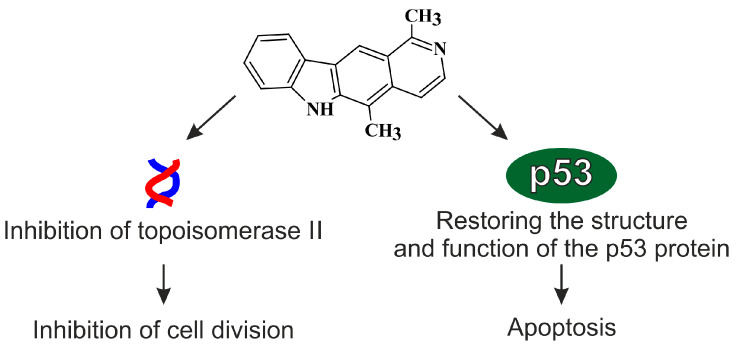
The mechanism of the antitumor action of olivacine.

The mechanism of action and antitumor properties of olivacine (presented in Figure 3) are similar to ellipticine and rely on direct interaction–intercalation in DNA and topo II activity [22,23]. It should be emphasized that some olivacine derivatives, e.g., compound S16020, showed a broad spectrum of antitumor activity and greater activity than ellipticine derivatives and doxorubicin [24,25].

### 4.1. Inhibition of the Function of Topoisomerase II (Topo II)

Previous studies indicate that olivacine derivatives bind to the topo II dissectible enzyme–DNA complex [71]. It is assumed that the stabilization of the cleavable complex “from the side” of the enzyme, i.e., drug interactions with the topo II complex, is more durable and effective, promising effective stabilization of the complex [76]. It has been shown that in addition to blocking topo II, olivacine derivatives also intercalate into DNA. Thus, the determination of the share of direct interactions of new derivatives with the nucleic acid and interactions with the topo II enzyme may help in understanding the mechanisms of genotoxic action of these compounds, especially in comparison with the standard topo II inhibitor that stabilizes the cleavable topo II–DNA complexes—etoposide, which it is a semi-synthetic derivative of podophyllotoxin [77].

### 4.2. p53 Protein

A promising direction of research in recent years in experimental oncology involves attempts to restore the structure and function of the p53 protein, which heralds the development of new therapeutic strategies, increasing the effectiveness of anticancer therapy. This is an important way to influence the death of cells through apoptosis, which in neoplastic disease is disturbed. Olivacine derivatives affect both the normal p53 protein and the wild-p53 protein [26,27].

## 5. Conclusions

In this review, we have described the alkaloid olivacine and its derivatives. Olivacine **1** has been known for 60 years, but all its properties are still not known. We know that it has been used against malaria, has anticancer properties, and inhibits *E. coli* bacteria. Its 9-methoxyolivacine derivative significantly inhibits the growth of *Mycobacterium tuberculosis*. Importantly, however, the olivacine derivatives show greater antitumor activity than doxorubicin and ellipticine. At the same time, they are less toxic than ellipticine and doxorubicin, which are currently used in cancer treatment. Based on in vitro and in vivo activity, it can be seen that derivatives with a methyl substituent in the N-6 position of the pyridine carbazole system have the best activity.

Interestingly, the derivatives that showed very good activity in in vitro tests did not translate into in vivo tests (compounds having a hydroxyl group in the C-9 position of the pyridocarbazole system). The best effect seems to be introducing a moiety in the C-9 position of the pyridocarbazole system, which in the body will hydrolyze to the OH group. Interestingly, olivacine derivatives show a stronger effect on the p53 protein than ellipticine, a protein responsible for DNA repair or cell apoptosis. Also of interest are olivacine derivatives **28** and **29**, which are active under hypoxic conditions but are very weakly active under aerobic conditions. These compounds may be precursors to drugs that act on anaerobic neoplasms and will not act on healthy cells. It seems that research on new olivacine derivatives may contribute to the development of new effective anticancer drugs.

## Figures and Tables

**Table 1:** In vitro and in vivo biological activity of compounds.

No.	Compound	Structure	In Vitro Cell Lines IC_50_ µM ± SD			In Vivo		Reference Number
			L1210	A549	MCF-7	Dose mg/kg (Cell Lines)	Therapeutic Effect %	
**1**	1,5-dimethyl-6*H*-pyrido[4,3-*b*]carbazole *olivacine*	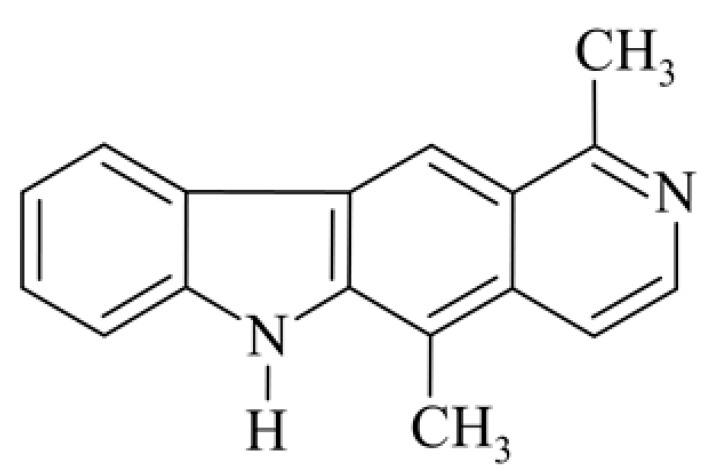	2.03			250 (L2110) 84.0 (L1220)	35 (ILS) 141 (T/C)	[35] [3]
**2**	9-hydroxy-1,5-dimethyl-6*H*-pyrido[4,3-*b*]carbazole *9-hydroxyolivacine*	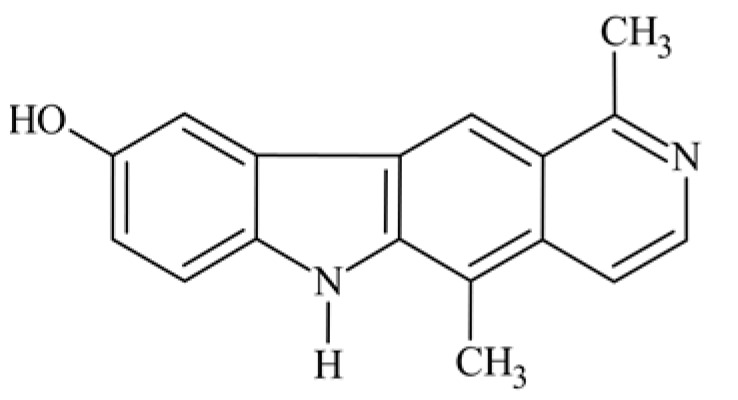	0.06					[35]
**3**	1,5-dimethyl-6*H*-pyridazino[4,3-*b*]carbazole	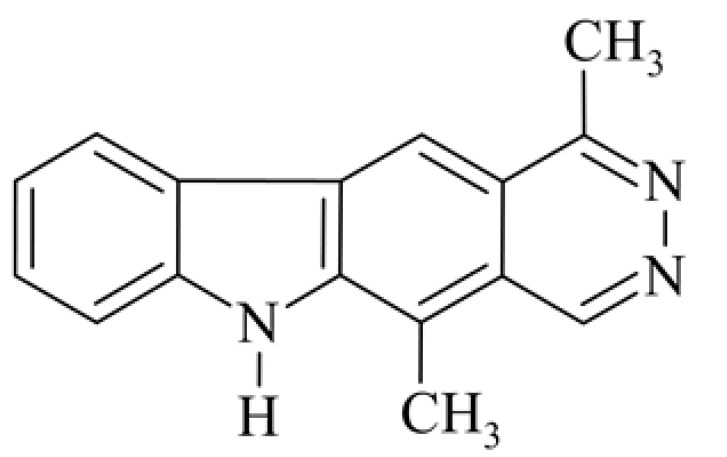	1.79		4.50			[24]
**4**	10-{[3-(diethylamino)propyl]amino}-6-methyl-5*H*-pyrido[3′4′:4,5]pyrrolo[2,3-g]isoquinoline *Pazellipticine (PZN)*	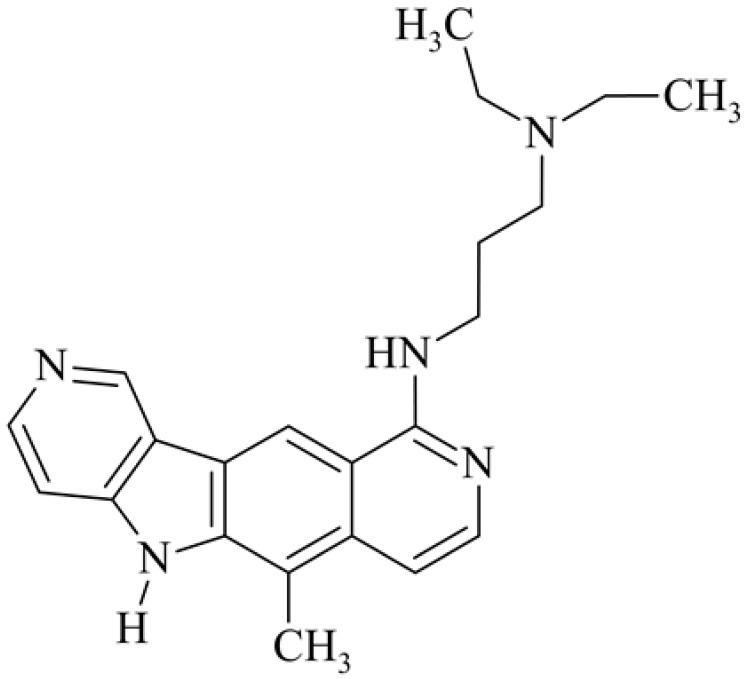	0.02			20 (L1210)	85 (ILS)	[36]
**5**	1-{[3-(diethylamino)propyl]amino}-9-methoxy-5-methyl-6*H*-pyrido[4,3-*b*]carbazole	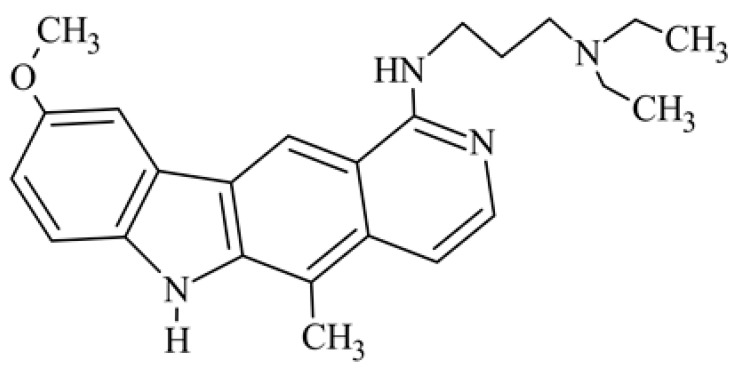	5			15 (L1210)	24.6 (ILS)	[36]
**6**	1-{[3-(diethylamino)propyl]amino]}-9-hydroxy-5-methyl-6*H*-pyrido[4,3-*b*]carbazole 1-{[3-(diethylamino)propyl]amino}-5-methyl-6*H*-pyrido[4,3-*b*]carbazol-9-ol	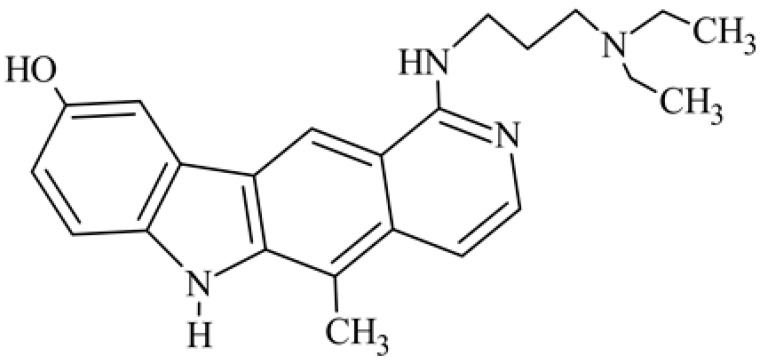	0.02			5 (L1210)	49.5 (ILS)	[37]
**7**	1-{{[3-(diethylamino)propyl]amino}methyl}-9-methoxy-5-methyl-6*H*-pyrido[4,3-*b*]carbazole	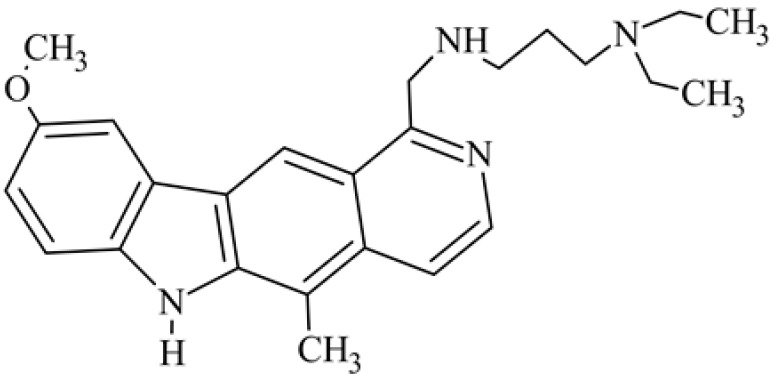	1			20 10 (L1210)	21 16 (ILS)	[38]
**8**	Pentanedioic acid mono{1-[2-(dimethylamino)ethyl]carbamoyl}-5,6-dimethyl-6*H*-pyrido[4,3-*b*]carbazol-9-yl] ester dihydrochloride S 30972-1	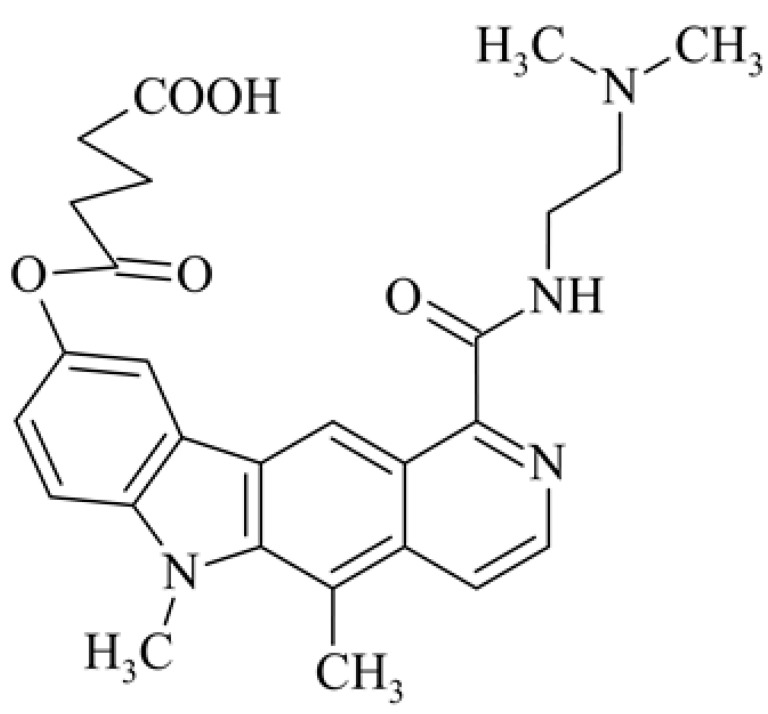 , 2 HCl	0.019			160-320 80 (P388)	246->590 427->582 (T/C)	[39]
**9**	9-hydroxy-1-{[2-(dimethylamino)ethyl]carbamoyl}-5,6-dimethyl-6*H*-pyrido[4,3-*b*]carbazole	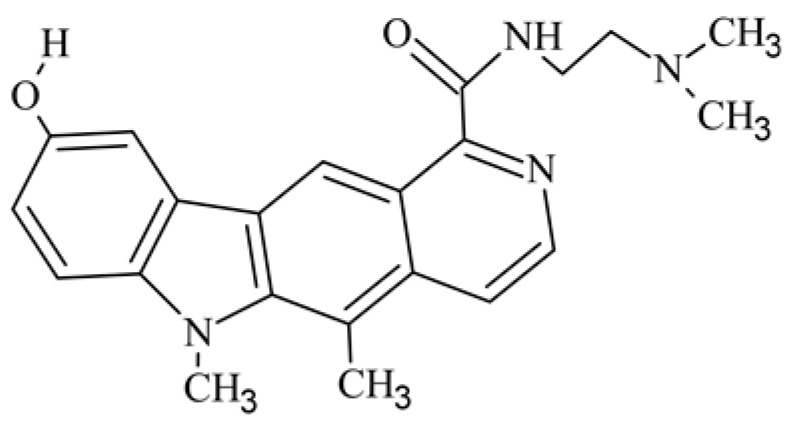	0.0041 ± 0.0006			90 (P388)	238 (T/C)	[40]
	9-hydroxy-5,6-dimethyl-6*H*-pyrido[4,3-*b*]carbazole-1-*N*-[2-(dimethylamino)ethyl]carboxamides S16020		0.0084 ± 0.0007	0.030 ± 0.004	0.075 ± 0.011	120 80 (P388)	301 >631 (T/C)	[41]
**10**	9-hydroxymethyl-1-{[2-(dimethylamino)ethyl)carbamoyl}-5,6-dimethyl-6*H*-pyrido[4,3-*b*]carbazole 9-hydroxymethyl-5,6-dimethyl-6*H*-pyrido[4,3-*b*]carbazole-1-*N*-[2-(dimethylami-no)ethyl]carboxamides	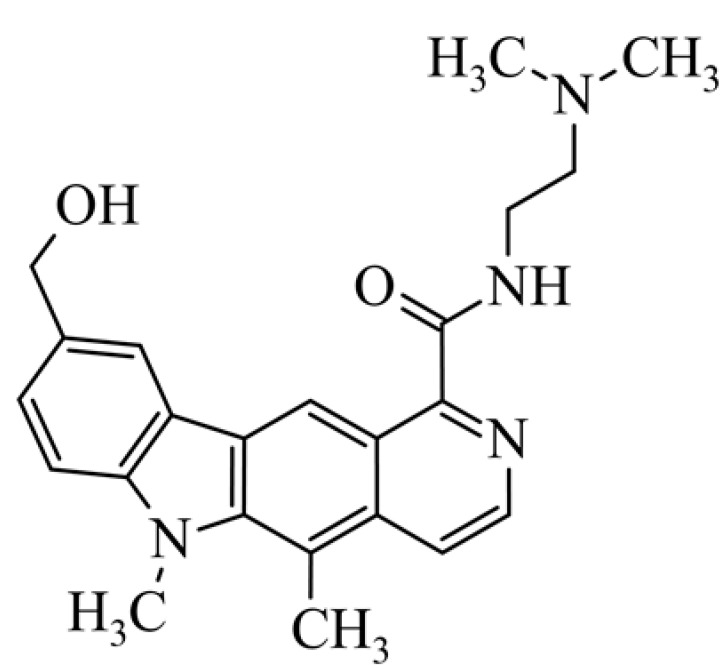		0.65 ± 0.3				[42]
**11**	5,6-dimethyl-1-{[2-(dimethylamino)ethyl]carbamoyl}-9-(*N*-methylcarbamoyloxymethyl)-6*H*-pyrido[4,3-*b*]carbazole (1-{[2-(dimethylamino)ethyl]carbamoyl}-5,6-dimethyl-6*H*-pyrido[4,3-b]carbazol-9-yl)methyl methylcarbamate	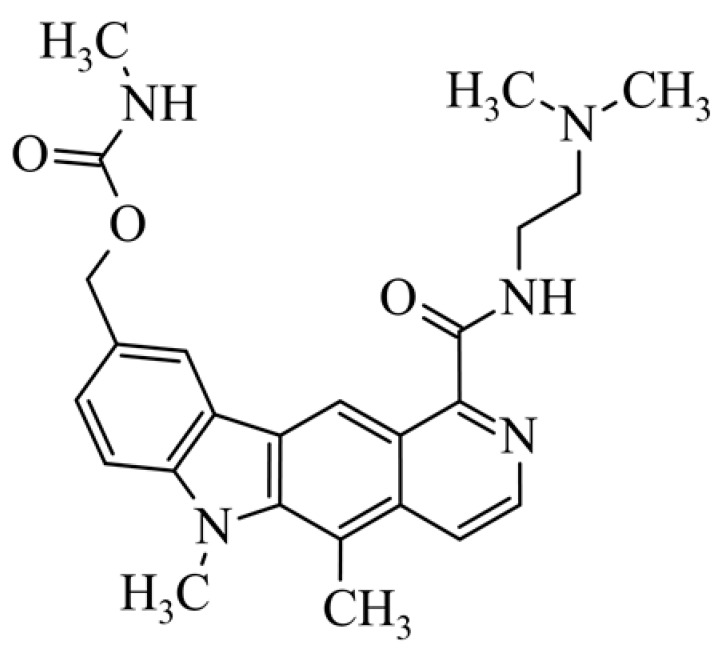		1.25 ± 0.29				[42]
**12**	9-methoxy-5,6-dimethyl-1-({[1-hydroxy-2-(hydroxymethyl)butan-2-yl]amino}methyl)-6*H*-pirydo[4,3-*b*]carbazole 2-ethyl-2-{[(5,6-dimethyl-9-methoxy-6*H*-pyrido[4,3-*b*]carbazole-1-yl)methyl]amino}propane-1,3-diol	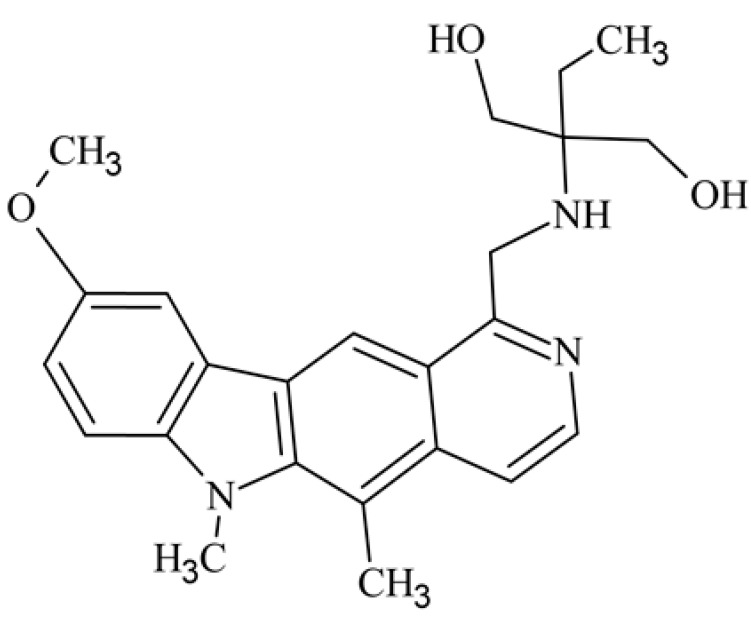		0.377 ± 0.159				[43]
**13**	9-hydroxy-5,6-dimethyl-1-{[(1-hydroxy-2-methylpropan-2-yl)amino]methyl} -6*H*-pirydo[4,3-*b*]carbazole 5,6-dimethyl-1-{[(1-hydroxy-2-methylpropan-2-yl)amino]methyl} -6*H*-pirydo[4,3-*b*]carbazol-9-ol	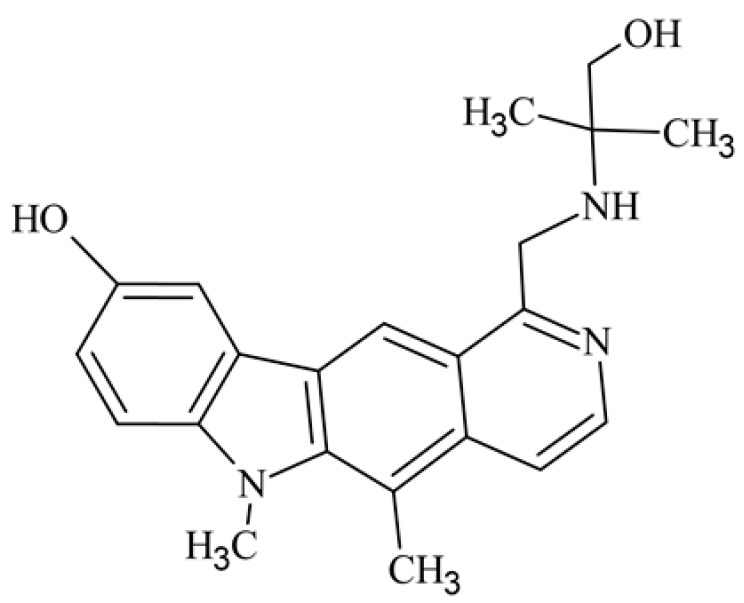		0.885 ± 0.071				[43]
**14**	9-hydroxy-1-hydroxymethyl-5,6-dimethyl-6*H*-pyrido[4,3-*b*]carbazole 1-hydroxymethyl-5,6-dimethyl-6*H*-pyrido[4,3-*b*]carbazol-9-ol	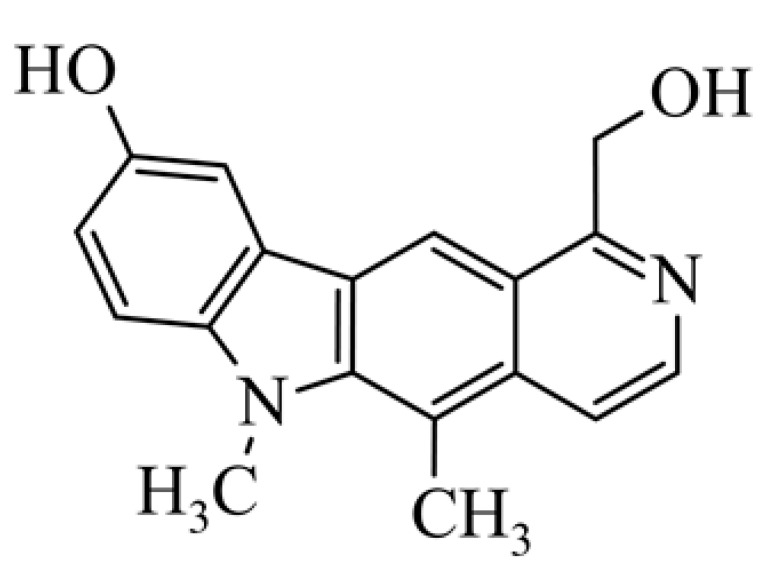		0.962 ± 0.52				[43]
**15**	7-hydroxy-10,11-dimethyl-*N*-[2-(dimethylamino)ethyl]- -10*H*-pyrimido[4,5-*b*]carbazole-4-carboxamide	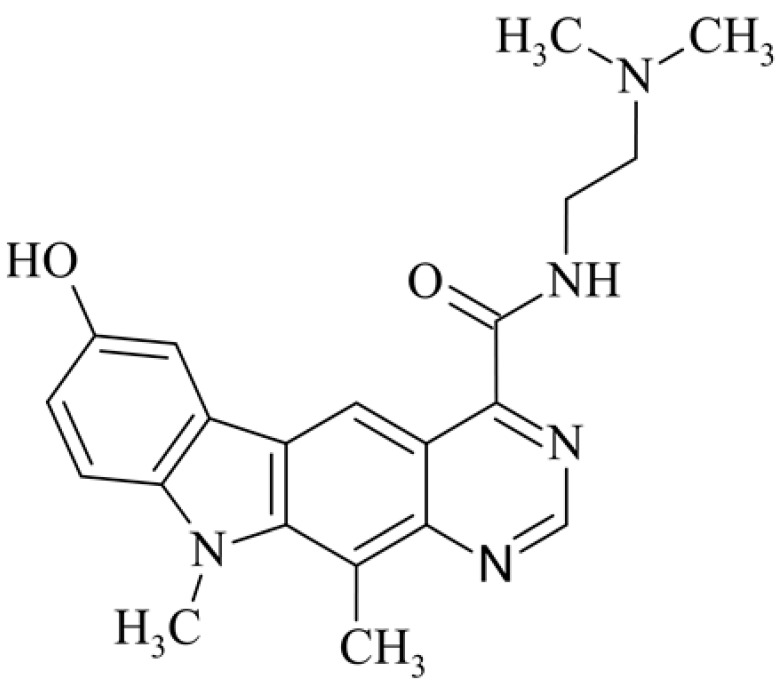	0.010					[44]
**16**	9-hydroxy-5,6-dimethyl-*N*-[2-(dimethylamino)ethyl]- 6*H*-pyrazino[2,3-*b*]carbazole-2-carboxamide	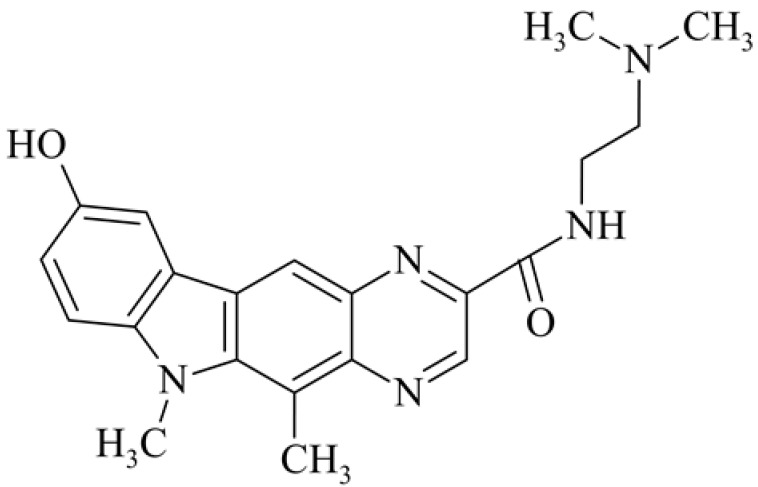	0.33					[44]
**17**	1-(4-aminophenyl)-9-hydroxy-5,6-dimethyl-6*H*-pyrido[4,3-*b*]carbazole 1-(4-aminophenyl)-5,6-dimethyl-6*H*-pyrido[4,3-*b*]carbazol-9-ol	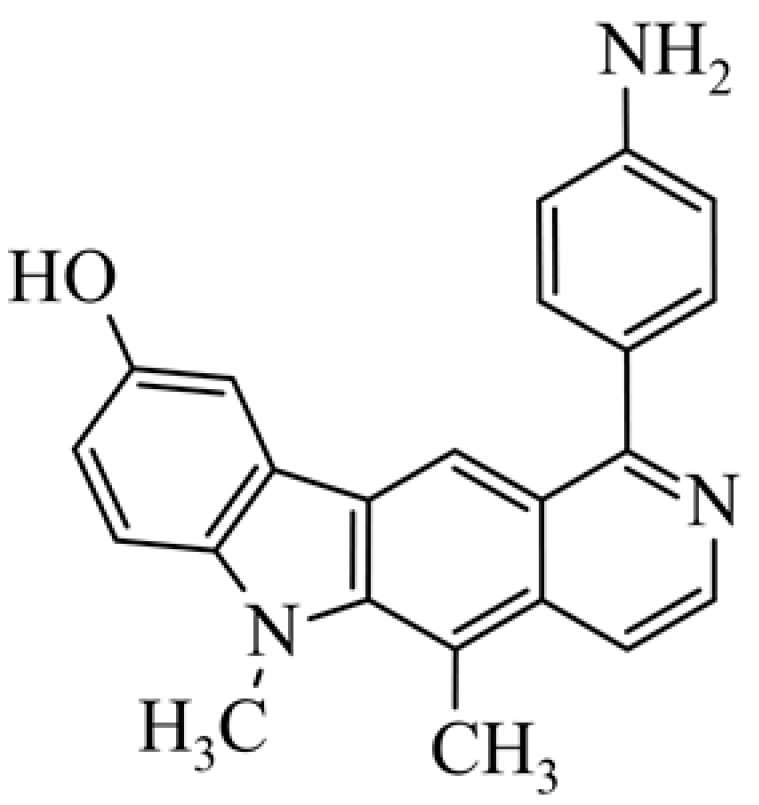	0.096 ± 0.031	0.51 ± 0.22				[45]
**18**	9-methoxy-5,6-dimethyl-1-{4-[*N*-[3-(dimethylamino)propyl]carbamoylphenyl}-6*H*-pyrido[4,3-*b*]carbazole *N*-[2-(dimethyla-mino)propyl]-4-(9-methoxy-5,6-dimethyl-6*H*-pyrido[4,3-*b*]carbazol-1-yl)benzamide	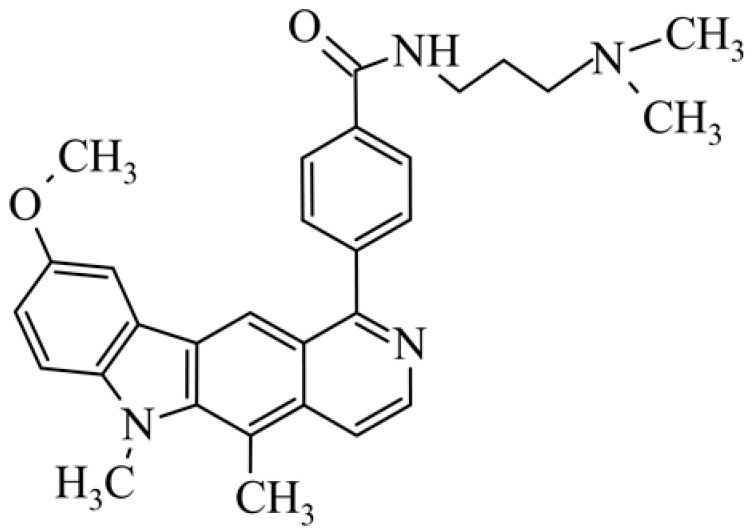	5.70 ± 0.33	4.30 ± 1.95				[46]
**19**	9-hydroxy-5,6-dimethyl-1-{4-[*N*-[2-(dimethylamino)ethyl]carbamoyl]phenyl}-6*H*-pyrido[4,3-*b*]carbazole *N*-[2-(dimethyla-mino)ethyl]-4-(9-hydroxy-5,6-dimethyl-6*H*-pyrido[4,3-*b*]carbazol-1-yl)benzamide	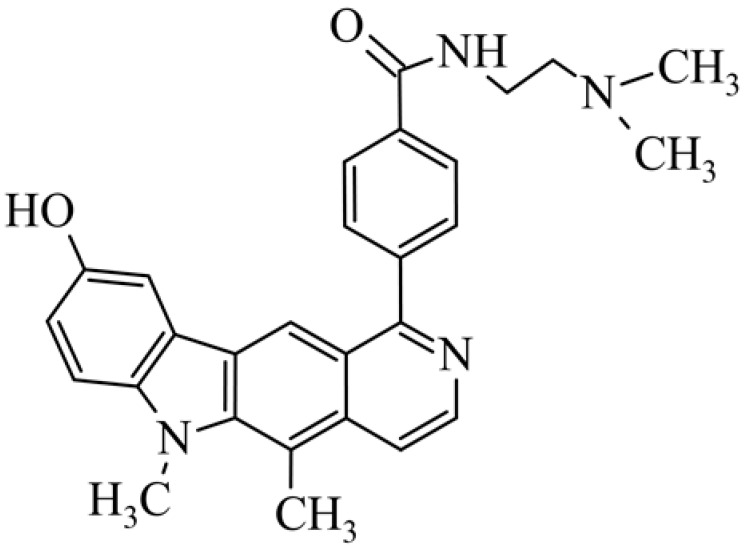	7.15 ± 2.76	8.19 ± 1.41				[46]
**20**	9-hydroxy-5,6-dimethyl-1-{3-[*N*-[2-(dimethylamino)ethyl]carbamoyl]phenyl}-6*H*-pyrido[4,3-*b*]carbazole *N*-[2-(dimethyla-mino)ethyl]-3-(9-hydroxy-5,6-dimethyl-6*H*-pyrido[4,3-*b*]carbazol-1-yl)benzamide	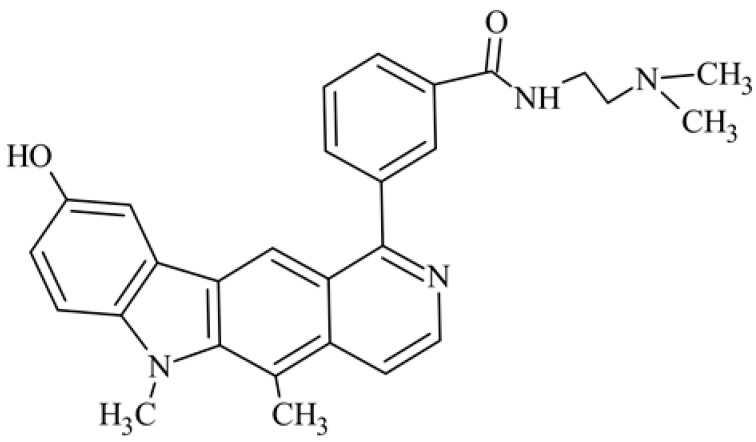	6.08 ± 3.96	8.25 ± 2.14				[46]
**21**	5,6-dimethyl-1-(4-nitro-phenyl)-6*H*-pyrido[4,3-*b*]carbazol-9-ol	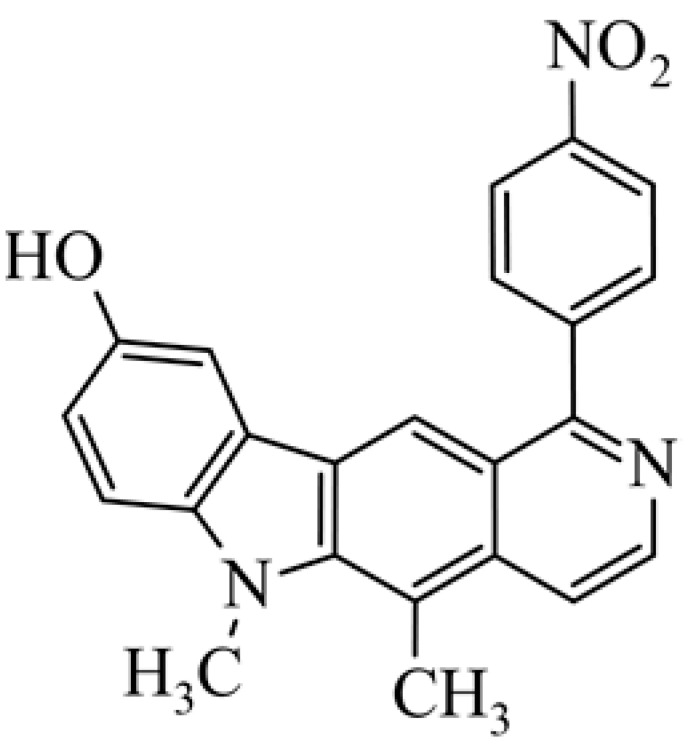		2.37 ± 3.41				[47]
**22**	1-(4-hydroxy-phenyl)-5,6-dimethyl-6*H*-pyrido[4,3-*b*]carbazol-9-ol	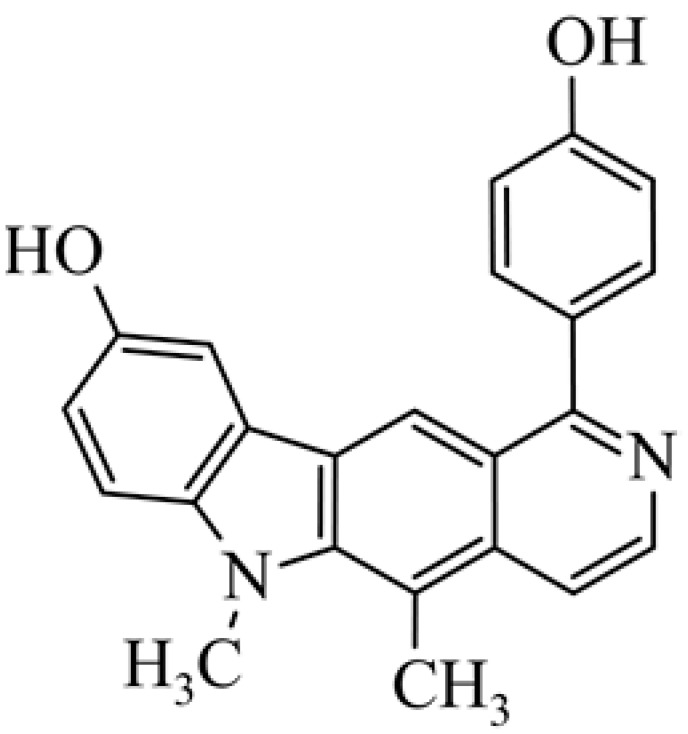		1.76 ± 0.05				[47]
**23**	6-(9-hydroxy-5,6-dimethyl-6*H*-pyrido[4,3-*b*]carbazol-1-yl)pyridine-2-carboxylic acid [2-(dimethylamino)ethyl]amide	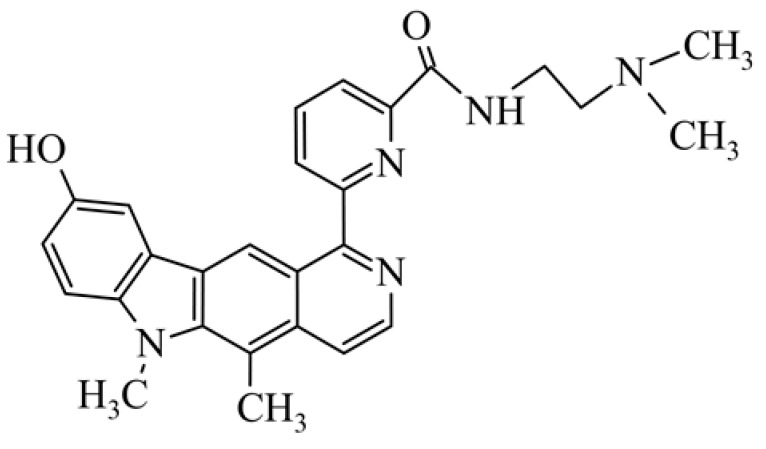	0.050 ± 0.011	0.095 ± 0.040	0.23 ± 0.020	40 (P388)	207 (T/C)	[41]
**24**	5,6-dimethyl-1-(6-methyl-pyridin-2-yl)-6*H*-pyrido[4,3-*b*]carbazol-9-ol	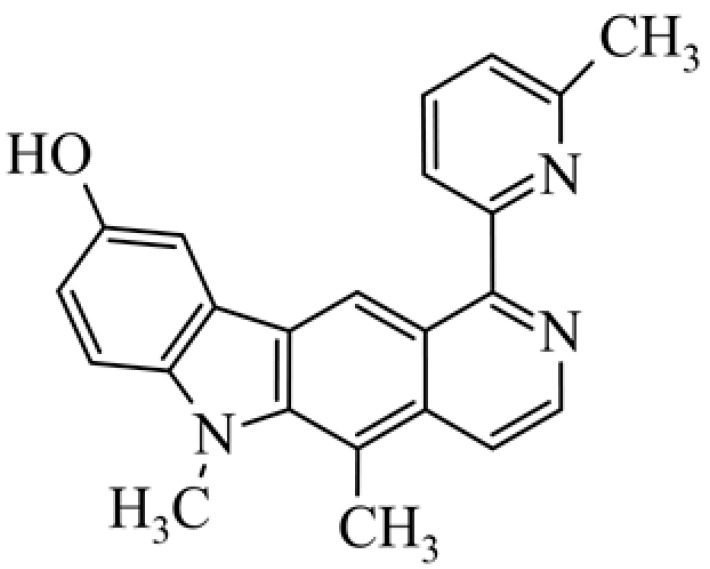	0.9	5.03				[42]
**25**	5-methyl-6-(2-dimethylamino-ethyl)-1-(6-methyl-pyridin-2-yl)-6*H*-pyrido[4,3*-b*]carbazol-9-ol	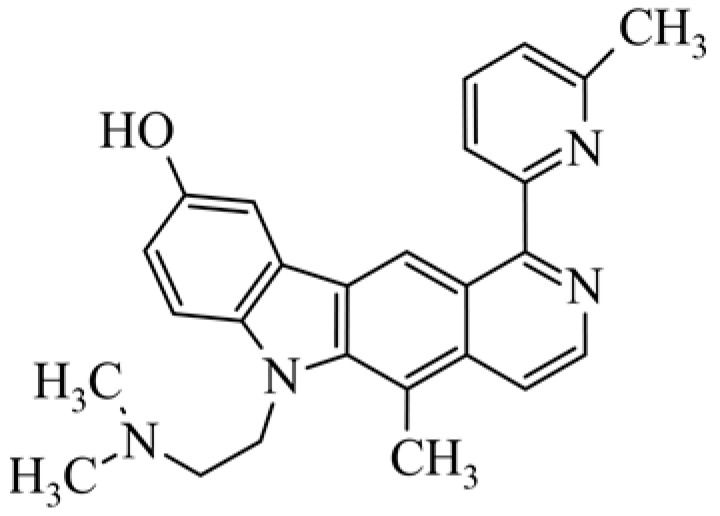	1.50	2.12				[48]
**26**	5,6-dimethyl-1-(2-methyl-pyridin-4-yl)-6*H*-pyrido[4,3-*b*]carbazol-9-ol	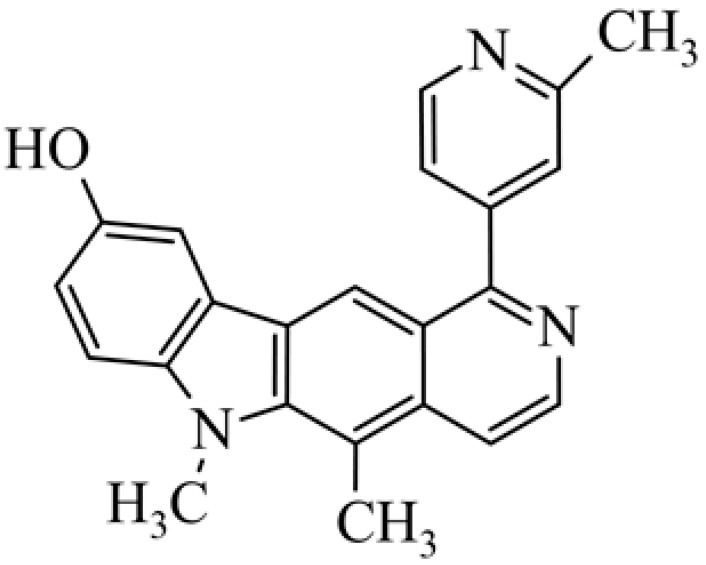	0.8					[49]
**27**	5,6-dimethyl-1-(6-methyl-pyridin-3-yl)-6*H*-pyrido[4,3-*b*]carbazol-9-ol	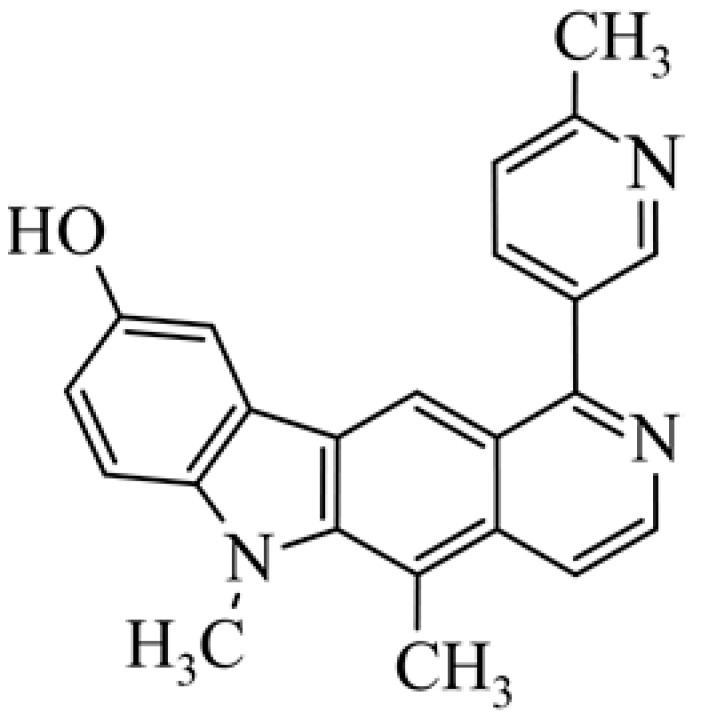	0.85					[50]
**28**	9-methoxy-5,6-dimethyl-1-(1-methyl-4-nitro-3*H*-imidazol-5-yl)-6*H*-pyrido[4,3-*b*]carbazole	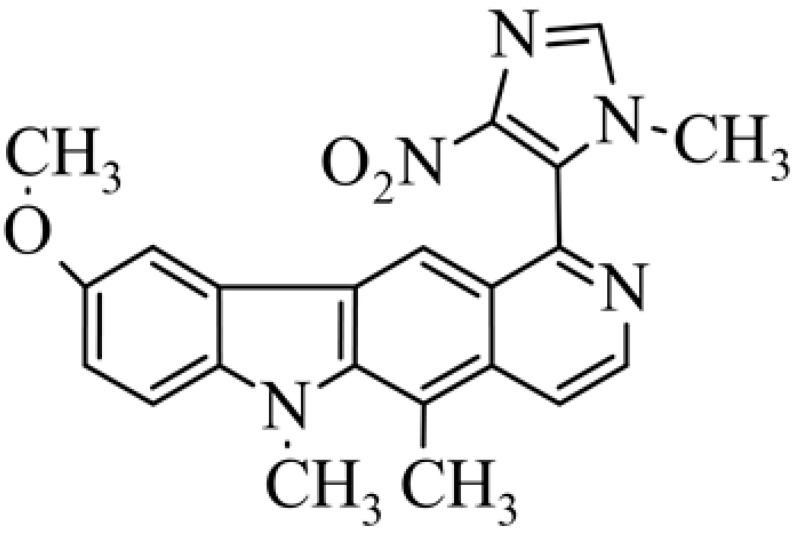		4.74± 1.30 (hypoxic 0.57 ± 0.033)	5.96 ± 1.10 (hypoxic 0.69 ± 0.053)			[51]
**29**	9-methoxy-5,6-dimethyl-1-(1-methyl-4-nitro-2*H*-pyrazol-5-yl)-6*H*-pyrido[4,3-*b*]carbazole	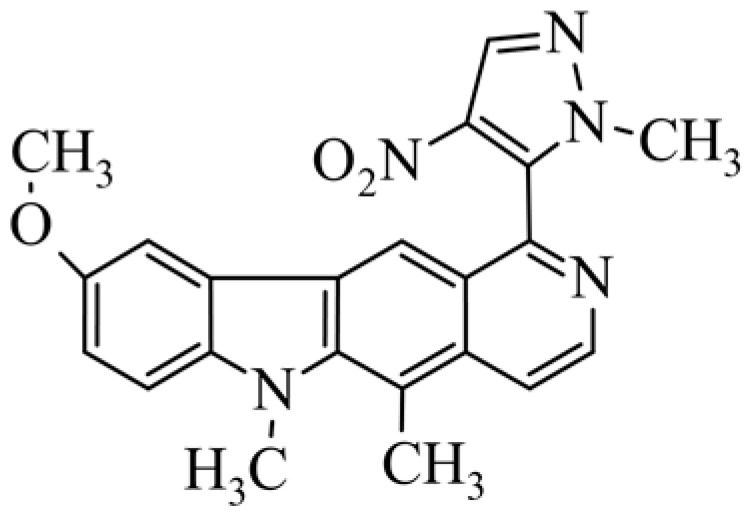		30.50 ± 5.05 (hypoxic 0.65 ± 0.019)	11.25 ± 3.42 (hypoxic 0.81 ± 0.045)			[51]

## Data Availability

Not applicable.
